# Asymptomatic intra-peritoneal rupture of hydatid cyst of the liver: case report

**DOI:** 10.1186/1756-0500-7-114

**Published:** 2014-02-26

**Authors:** Anass M Majbar, Mehdi Aalala, Mouna Elalaoui, Farid Sabbah, Mohamed Raiss, Abdelmalek Hrora, Mohamed Ahallat

**Affiliations:** 1 Clinique Chirurgicale C. Ibn Sina hospital, Rabat, Morocco

**Keywords:** Echinococcosis hepatic, Albendazole, Rupture

## Abstract

**Background:**

Intra-peritoneal rupture of hydatid cyst is a rare complication and there is no consensus about its treatment.

**Case presentation:**

The reported case concerns a 25 years old female patient who had been complaining for four months from a moderate pain in the right upper quadrant. No clinical or biological signs of sepsis or allergic reactions were witnessed. Ultrasound and CT examinations showed a multilocular hepatic cyst in addition to multiple unilocular cysts in the abdomen. The suspected diagnosis was hepatic and peritoneal HC and a surgical treatment was scheduled four weeks later. Surgical exploration showed a large ruptured HC on the left lobe of the liver, with daughter cysts in the peritoneal cavity. Left lobectomy of the liver with complete ablation of all daughter cysts and a wide peritoneal lavage were performed. For the three months following the surgery, Albendazole had been given to the patient. No recurrence occurred after four years of follow-up.

**Conclusion:**

Intra-peritoneal rupture of liver HC could be asymptomatic. This case showed that in some cases, occurrence of complications is not systematic. This suggests that urgent surgical treatment is not always mandatory in the absence of alarming signs. Well-conducted medical treatment would reduce the risk of occurrence of secondary peritoneal hydatidosis.

## Background

Intra-peritoneal rupture is a serious turning point in the evolution of hepatic hydatid disease. It is a rare complication and there is still a debate in the literature about a reliable treatment. The reported case concerns a patient suffering from an asymptomatic intra-peritoneal rupture of a hydatid cyst in the left liver, which was discovered intra-operatively, highlighting the implications of this atypical mode of presentation.

## Case presentation

**Figure 1:**
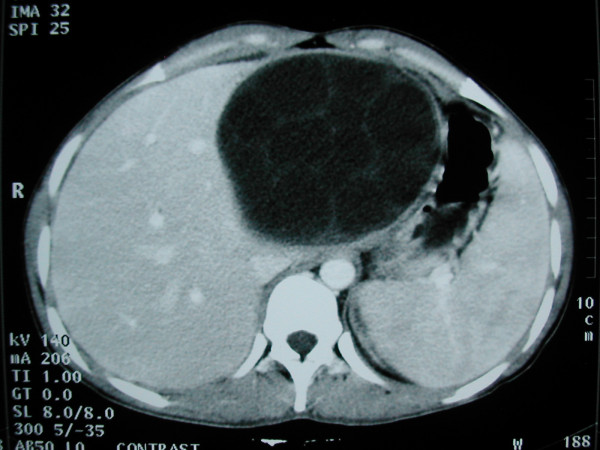
Hydatid cyst of the left liver with typical multivesicular image.

**Figure 2:**
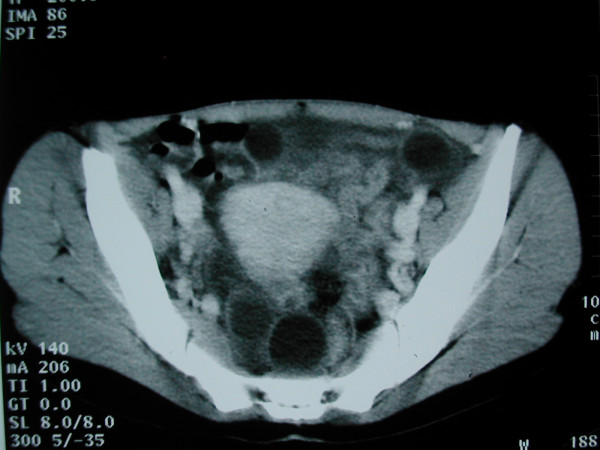
Daugther cysts seen as unilocular cysts in the peritoneal cavity.

**Figure 3:**
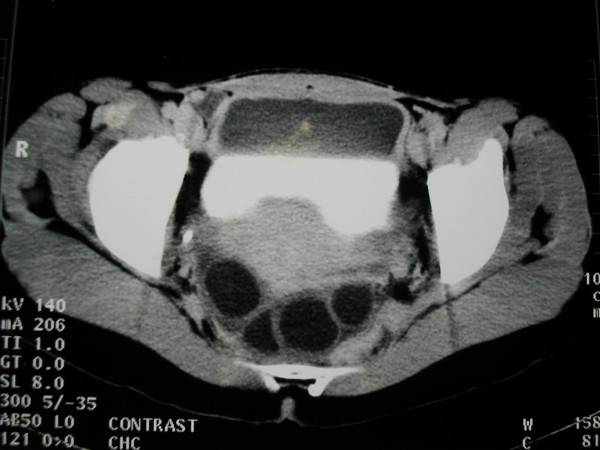
Unilocular cysts in the pelvis.

A twenty-five years old female patient was referred to our unit after showing symptoms of abdominal hydatid disease. Her medical history was uneventful and she had been complaining for four months from a moderate pain of the right upper quadrant. Clinical examinations revealed an absence of fever or allergic symptoms and a soft abdomen. However, there was a firm painless mass in the right upper quadrant. Abdominal ultrasonography (US) showed a cystic mass of the left hepatic lobe, corresponding to a type II hydatid cyst (HC). In addition to this, it also showed a multilocular cystic in the pelvis. CT scan revealed (Figures 1, 2 and 3) a single multilocular hepatic cyst of 11 cm in segments II, III and IV and multiple unilocular cysts in the right abdominal quadrant, hypogatric area and the pelvis. The chest radiography was normal. The laboratory work-up included normal standard preoperative tests and positive hydatid serology. The suspected diagnosis was hepatic and peritoneal HC. Therefore, a surgical treatment was scheduled four weeks after the CT-scan. Before admission for surgery, there were no changes in clinical (absence of abdominal tenderness) or biological status (normal white cell count). Surgical exploration showed a large ruptured HC of the left lobe of the liver, with multiple daughter cysts in the peritoneal cavity (right sub-phrenic area, pelvis, paracolic gutters). A left lobectomy of the liver, an ablation of all the daughter cysts in the peritoneal cavity and a wide peritoneal lavage were performed. The postoperative period was uneventful. The patient was assigned to a medical therapy (Albendazole: 15 mg/kg/day) for three months. No recurrence occurred after four years of clinical and radiologic (US) follow-up.

### Comments

Intra-peritoneal rupture is a rare complication of hepatic hydatid disease even in endemic areas. Its incidence varies in the literature from 1.75 to 8.6% [1-3]. The rupture could be spontaneous or more often following a trauma. Akcan et al. reported that young age, cyst diameter superior to 10 cm and superficial position are factor risks for a rupture of the HC of the liver [4].

Most frequently, the rupture in the peritoneum is symptomatic, with constant abdominal symptoms [2,5] (abdominal pain, vomiting, abdominal tenderness and/or rebound), as well as marked allergic symptoms in up to 25% of the cases [3] (cutaneous rash, urticaria, anaphylactic shock). These symptoms are related to complications (peritonitis, anaphylactic shock) secondary to intra peritoneal rupture, which are reported to occur in up to 100% of patients [6]. However, as shown in this case, this evolution is not systematic. Intra peritoneal rupture was asymptomatic, probably because no abdominal or anaphylactic complications occurred, which explains the late diagnosis, made several weeks after the rupture, during the surgery.

There is no consensus regarding the treatment in the case of an intra-peritoneal rupture. The majority of authors recommend an emergency surgical treatment [6] due to the risk of peritonitis that could be fatal to the patient [7]. But, as demonstrated here, the patient had a delayed surgical treatment with favourable outcomes. After the diagnosis of the intra-peritoneal rupture of a HC, prompt care with close monitoring must be initiated due to the risk of anaphylaxis and the occurrence of abdominal complications. The existence of sepsis, acute abdomen, intra-peritoneal bile leakage, or shock imposes an urgent surgical intervention. However, in the absence of complications, surgery may be postponed until a more appropriate time [8].

The recurrence rate following an intra-peritoneal rupture of the liver HC could reach 21% [5]. The use of Albendazole to prevent secondary peritoneal echinococcosis is recommended by all the authors, but there is no consensus regarding the duration of the treatment. A minimum duration of three months is advised [2]. In this case, the patient received medical treatment for three months, and did not show a recurrence after four years of follow-up.

## Conclusion

The intra-peritoneal rupture of liver hydatid cysts could be asymptomatic and the occurrence of complications is not systematic. This suggests that in case of early diagnosis of intra peritoneal rupture, urgent laparotomy should not be mandatory in the absence of alarming signs, and could be performed after correct preparation. Well-conducted medical treatment would reduce the risk of occurrence of secondary peritoneal hydatidosis.

## Consent

Written informed consent was obtained from the patient for publication of this Case report and any accompanying images. A copy of the written consent is available for review by the Editor of this journal.

## Competing interests

The authors declare that they have no competing interests.

## Authors’ contributions

MA and AM wrote the paper, HA provided data; AM, RM, EM, SF and HA revised the manuscript. All authors read and approved the final manuscript.
